# 2222. A study of the role of trimethoprim-sulfamethoxazole in opportunistic infection prevention in lupus patients taking low-level immunosuppressive treatment: An open-labeled, randomized controlled trial

**DOI:** 10.1093/ofid/ofad500.1844

**Published:** 2023-11-27

**Authors:** Paopat Munthananuchat, Pintip Ngamjanyaporn, Prapaporn Pisitkun, Porpon Rotjanapan

**Affiliations:** Faculty of Medicine Ramathibodi Hospital, Mahidol University, Bangkok, Krung Thep, Thailand; Faculty of Medicine Ramathibodi Hospital, Mahidol University, Bangkok, Krung Thep, Thailand; Faculty of Medicine Ramathibodi Hospital, Mahidol University, Bangkok, Krung Thep, Thailand; Faculty of Medicine Ramathibodi Hospital, Mahidol University, Bangkok, Krung Thep, Thailand

## Abstract

**Background:**

Individuals with lupus are at risk of opportunistic infections (OIs), especially pneumocystis pneumonia and nocardiosis. The current recommendation to prevent OIs is to take trimethoprim-sulfamethoxazole (TMP/SMX) regularly while on treatment for lupus. However, there has yet to be a standard recommendation on whether TMP/SMX prophylaxis is necessary and when prophylaxis can be discontinued.

**Objectives:**

To assess the role of TMP/SMX in primary prophylaxis of OIs and to study the incidence of TMP/SMX sensitive OIs and the adverse events of TMP/SMX in the real-world setting among lupus patients taking low-level immunosuppressive therapy.

**Methods:**

An open-label, randomized controlled trial was conducted among lupus patients taking low-level immunosuppressive at Ramathibodi Hospital between May 2021 and April 2023. The patient's demographic and relevant data were retrieved. The patients were randomized to receive or not receive TMP/SMX in a 1:1 ratio. The dose of TMP/SMX was adjusted based on renal function. The incidence of TMP/SMX-sensitive OIs was monitored until one year from enrollment, and the adverse events were assessed.

**Results:**

The study was terminated prematurely due to an exceedingly high rate of adverse events secondary to TMP/SMX use. The analysis was made for the enrolled 138 lupus patients at six months, and OIs were monitored until one year. The mean ages were 45.4 in both groups. Most patients (98.4%) had SLEDAI-2K score < 4. The doses of immunosuppressive therapy were adjusted in six patients due to active disease and therefore excluded from the study. No TMP/SMX sensitive OIs documented in 114 lupus patients who completed follow-ups over nine months since enrollment. Eight from 10 patients had grade 1, whereas 2/10 patients had grade 3 adverse drug reactions, and all declined to resume prophylaxis. There was no mortality in the study.

**Conclusion:**

The incidence of TMP/SMX-sensitive OIs among lupus patients taking low-level immunosuppressive was not a significant concern after a one-year follow-up, and high rates of adverse events with TMP/SMX prophylaxis were documented. Therefore, TMP/SMX primary prophylaxis may not be justified in this population.

**Disclosures:**

**All Authors**: No reported disclosures

